# P-2077. Carbapenemase Resistance Detection by Direct Xpert Carba-R Assay on Flagged Blood Cultures

**DOI:** 10.1093/ofid/ofae631.2233

**Published:** 2025-01-29

**Authors:** Akansha Didwania, Manasvini Bhatt, Parul Kodan, Binit Kumar Singh, Sowjanya Perumalla, Sarita Mohapatra, Naveet Wig, Manish Soneja

**Affiliations:** All India Institute of Medical Sciences, New Delhi, Delhi, India; ALL INDIA INSTITUTE OF MEDICAL SCIENCES,NEW DELHI, New Delhi, Delhi, India; All India Institute of Medcial Sciences, Delhi, Delhi, India; All India Institute of Medical Sciences, New Delhi, New Delhi, Delhi, India; All India Institute of Medical Sciences, New Delhi, Delhi, India; All India Institute of Medical Sciences, New Delhi, New Delhi, Delhi, India; All India Institute of Medical Sciences, New Delhi, Delhi, India; All India Institute Of Medical Sciences, Delhi, Delhi, India

## Abstract

**Background:**

Early detection of carbapenem-resistant gram negative bacilli(CR-GNB) is critical in patients with sepsis. Delay in diagnosis leads to higher mortality. We evaluated the performance of the Xpert® Carba-R assay (Cepheid, Sunnyvale, CA, USA) directly on flagged blood culture samples in patients with sepsis.

**Fig 1:**
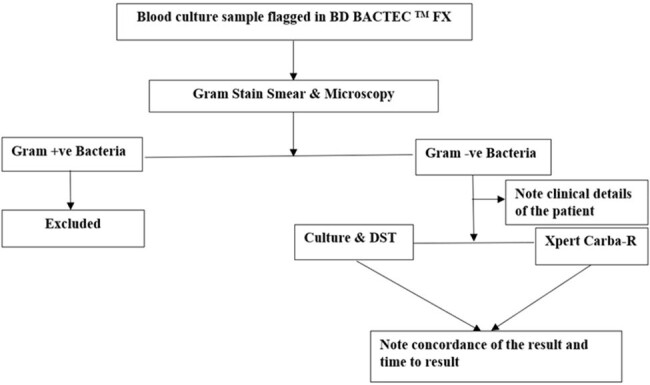
Workflow

**Methods:**

Patients with suspected sepsis with GNB on flagged blood culture were recruited. Xpert® Carba-R assay was performed directly on the positive sample. Their diagnostic performance was compared with culture and drug susceptibility testing (DST)(VITEK-2) and time to results were noted and compared.

**Table 1:**
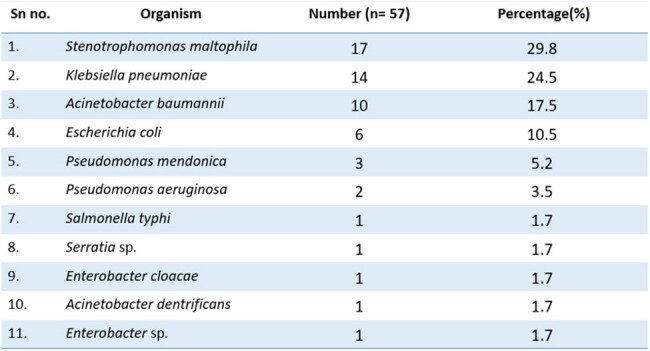
Organism profile of patients with sepsis (n=57)

**Results:**

In the study, 57 patients were included; *Stenotrophomonas maltophila* (n=17; 29.8%) was found to be the predominant isolate, which was excluded from analysis (being intrinsically resistant to carbapenems). This was followed by *Klebsiella pneumoniae* (n=14 ; 24.5%), *Acinetobacter baumannii* (n=10 ;17.5%), *Escherichia coli* (n=6;10.5%), *Pseudomonas mendonica* (n=3; 5.2%), *Pseudomonas aeruginosa* (n=2;3.5%) and 1 each of *Salmonella typhi*, *Serratia* sp., *Enterobacter cloacae*, *Acinetobacter dendrificans*, and *Enterobacter* sp. Xpert® Carba-R positivity was seen in 23/40 isolates (57.5%), and CR GNB was found in 25/40 isolates (62.5%) on routine DST. Discordant results were seen in 6/40 isolates (15%). Overall sensitivity, specificity, positive predictive value, negative predictive value, positive likelihood ratio, and negative likelihood ratio of Xpert® Carba-R compared to routine DST (Disk diffusion method and VITEK-2) were 84%, 86.6% , 91.3%, 76.47 %, 6.30 and 0.18 respectively. On genomic analysis, most common CR gene expression was co-expression of NDM and OXA-48 [10/23 (43.5%)], followed by NDM [9/23 (39.1%)] and OXA-48 [4/23 (17.4%)]. The median time to obtain Carba-R result was 44 hours compared to 97 hours for detection of carbapenem resistance in routine DST, giving a 53-hour lead time.

**Table 2:**
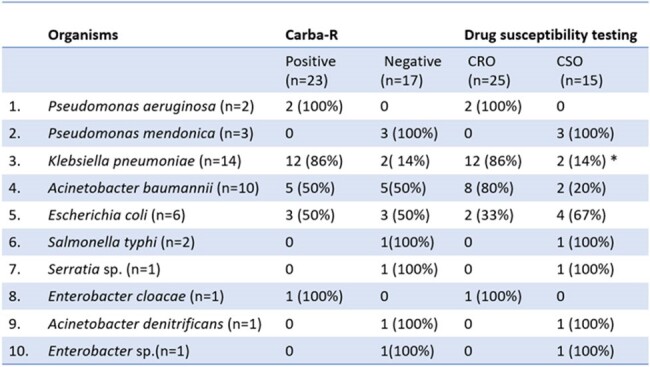
Comparison of direct Xpert® Carba-R and DST ( disc diffusion method and Vitek-2) in various organisms

**Conclusion:**

Xpert® Carba-R was found to have a good diagnostic performance for detection of carbapenem resistance when performed directly on positive blood culture samples with substantial time advantage over conventional methods.

**Fig 2:**
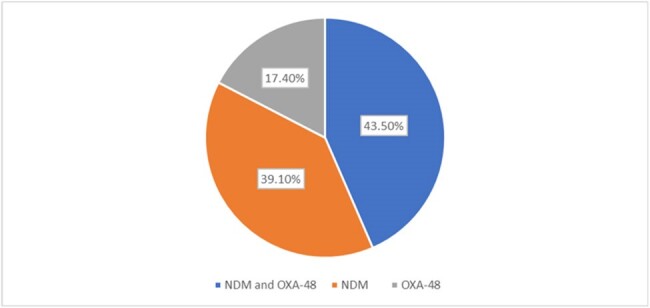
Distribution of Carbapenem resistant genes *Two discordant sample CRO- Carbapenem resistant organism CSO- Carbapenem sensitive organism

**Disclosures:**

All Authors: No reported disclosures

